# Removal efficiency of pharmaceuticals during the wastewater treatment process: Emission and environmental risk assessment

**DOI:** 10.1371/journal.pone.0331211

**Published:** 2025-09-24

**Authors:** Paulina Chaber-Jarlachowicz, Barbara Gworek, Radosław Kalinowski

**Affiliations:** 1 Department of Environmental Chemistry and Risk Assessment, Institute of Environmental Protection – National Research Institute, Warsaw, Poland; 2 RADIKAL Science Consultancy, Warsaw, Poland; National Research and Innovation Agency, INDONESIA

## Abstract

The release of pharmaceuticals into the environment is a major concern. These compounds enter waterways through the effluent of wastewater treatment plants (WWTPs). However, most WWTPs using mechanical-biological processes based on activated sludge (CAS) are unable to effectively remove pharmaceuticals. Consequently, pharmaceuticals end up in surface water, seawater and groundwater. While some pharmaceuticals break down or degrade, most remain unchanged and eventually become persistent in the environment, retaining their biological activity even at extremely low concentrations. This study aimed to investigate the occurrence, removal efficiency, environmental discharge and ecological risks of selected pharmaceuticals in municipal WWTPs. Samples were collected from six WWTPs serving over 200,000 people. Concentrations of pharmaceuticals were analysed using the LC-MS/MS method. Removal efficiency was assessed using mass balance calculations for pharmaceuticals in the influent, effluent and sludge. The potential ecological risk posed by individual pharmaceuticals was then evaluated based on the risk quotient (RQ). Concentrations of pharmaceuticals ranged from 7 ng/L to 1,019 ng/L in the influent, from 9 ng/L to 2,266 ng/L in the effluent and from 8.5 μg/kg to 406 μg/kg dw in the sewage sludge. All six WWTPs released pharmaceuticals into the environment. Naproxen, salicylic acid and ketoprofen were the only compounds effectively removed during treatment. Fluoxetine and loratadine posed the greatest risk to aquatic organisms. These findings will lay the groundwork for further research into the inactivation of pharmaceutical active substances and their metabolites in sewage and sludge.

## Introduction

In recent years, there has been increasing awareness of the presence of excessive amounts of chemicals used in both industry and households in the environment These natural or man-made substances are known as ‘emerging contaminants’ (ECs) and are recognised as posing potential or actual threats to human health, aquatic life, and the environment. Many of them are not yet subject to comprehensive regulations or established health standards. Examples of ECs include pharmaceuticals, personal care products, industrial chemicals, pesticides and microplastics [1–3] These contaminants can enter the environment in various ways, including through industrial discharges, agricultural runoff, wastewater treatment plant effluent, and everyday consumer products. According to Archer at al., emerging contaminants represent a growing concern due to the potential risks associated with them and the limited knowledge we have about their behaviour and impact [3]. Continued research, monitoring, and the development of effective mitigation strategies are crucial to addressing this challenge [1–4]. A significant proportion of ECs consists of pharmaceutical products applied in medicine, veterinary medicine, animal husbandry and fish farming The consumption of pharmaceuticals has increased exponentially, with the current tonnage measured in hundreds. This increase can be attributed directly to the ongoing development of medicine and veterinary science, which has resulted in the release of these substances into the environment [5–8]. According to Puckowski at al, pharmaceuticals are designed to have specific biological effects at low concentrations. Their levels in the environment range from ng/L to μg/L. Their continued release into the environment can lead to long-term exposure and biomagnification, as toxicity can be enhanced by passing through trophic levels in the food chain. Although some pharmaceuticals break down or degrade upon consumption or release into the environment, most of them remain unchanged and eventually become persistent in the environment [4]. Gworek at al state that many of these chemicals remain bioactive even at extremely low concentrations. excretion from the body or disposal to landfills. When mixed together, they can have unpredictable biochemical interactions. They also tend to accumulate in the food chain, which can have a negative impact on the aquatic organisms and the human health [9] The volume of data supporting the presence of pharmaceutical residues in the environment (mainly in water, soil and sediment) is growing all the time [1,4,10–14]

According to Sim at al., the main sources of pharmaceuticals in the environment are wastewater from municipal treatment plants, livestock farms, hospitals and pharmaceutical manufacturers. The degradation rate of these substances is being exceeded by their release. The A significant concern pertains to the uncontrolled release of pharmaceuticals into the environment, predominantly via municipal and industrial wastewater from wastewater treatment plants (WWTPs) [8]. Research indicates that the highest concentrations of pharmaceuticals can be found in raw wastewater entering municipal wastewater treatment plants (WWTPs) [1,2,5,7,9,13–18]. The existing literature indicates that most wastewater treatment plants using mechanical-biological processes based on conventional activated sludge (CAS) lack the ability to remove pharmaceuticals effectively [1,5,9,12,16,19–25]. Giebułtowicz et al. state that the treatment of municipal wastewater usually involves mechanical and biological methods. Pharmaceutical removal efficiency depends on the substance and its interaction with suspensions, and is typically low. Higher efficiencies are achieved at the biological stage, where activated sludge is employed. Reductions in pharmaceutical compounds are achieved through adsorption on sludge flocs and biodegradation [24]. The removal efficiencies of pharmaceuticals from the aqueous phase are calculated based on the difference between the concentration of pharmaceuticals in the influent and effluent and are expressed as a percentage. In many studies, the rate of removal efficiency adopts negative values [21,22,26–28].

Numerous studies have investigated the removal efficiency, emissions, and risk assessment of pharmaceuticals in wastewater in various countries worldwide. Research conducted in a number of countries, including Canada [29], Korea [27], China [6,16,30], Algiers [18], and several European countries, Ireland [31], Italy [32] Spain [5], has indicated the occurrence of pharmaceuticals in both effluent and influent. In certain instances, negative values of removal efficiency have also been documented. Loos at al asses the occurrence of as many as possible polar organic chemical contaminants included pharmaceuticals in WWTP effluents of 90 Western European availableEU Member States [33]. The samples came from Austria (number of samples: 6), Belgium (18), Czech Republic (7), Cyprus (2), Finland (6), France (5), Germany (2), Greece (2), Hungary (2), Ireland (2), Italy (2), Lithuania (3), Netherlands (11), Portugal (2), Slovenia (1), Spain (3), Sweden (11), and Switzerland (5). The findings of the study indicate an elevated frequency of detection for pharmaceuticals, ranging from 50% to 90%. However, research focusing on the presence of pharmaceuticals in wastewater in Central and Eastern Europe is scarce. This issue therefore needs to be addressed. Furthermore, the presence of pharmaceuticals in the wider environment is a subject that is largely unmonitored in Central and Western Europe. For example, as shown in the case of Poland, there is little existing literature on pharmaceuticals in wastewater The majority of these studies are conducted at the local level and/or for individual WWTPs [7,24–26,34–36]. According to the Statistical Yearbook of the Republic of Poland, the average annual figure for pharmaceutical sales in Poland was PLN 14,568 million, equivalent to 25,784 tonnes of pharmaceutical products [37] Poland is distinguished by its comparatively limited water resources and substantial population. Gworek et al estimated that over 82% of the water utilised by the Polish economy is sourced from surface water, with a further 16% derived from groundwater and a minimal percentage, amounting to approximately 1%, discharged from mines. Surface water resources are the primary source of drinking water. The economic utilisation of water and the function of rivers, streams, and lakes as recipients of wastewater have a substantial impact on water quality and quantity [4,9]. Consequently, it is imperative to undertake a comprehensive investigation into the occurrence, removal efficiency, emission, and risk assessment of the most prevalent pharmaceuticals in Polish conventional WWTPs. Furthermore, there is an urgent necessity for research to be conducted into the presence of pharmaceuticals in wastewater and sewage sludge, with a particular emphasis on wastewater treatment processes that demonstrate analogous characteristics. Giebutowicz et al. estimated that the majority of wastewater treatment plants (WWTPs) in Poland use the conventional activated sludge (CAS) system [24].The primary objective of the present study was to estimate the removal efficiency and emission of pharmaceuticals to the environment. This was achieved by calculating the daily mass load of pharmaceuticals during the mechanical-biological process of wastewater treatment in municipal wastewater treatment plants using conventional activated sludge (CAS). The results of the concentration of pharmaceuticals in the treated effluent were then used to estimate the potential risk they could pose to aquatic organisms. Samples were collected from six wastewater treatment plants (WWTPs) located in Poland’s six largest agglomerations, with a population equivalent (PE) of over 200,000.The removal efficiency assessment was based on mass balance calculations of pharmaceuticals in the influent, effluent and sludge. The underlying research hypothesis was that in the context of large urban agglomerations, conventional municipal wastewater treatment plants would demonstrate an ineffective capacity to remove the pharmaceuticals under investigation from the treated effluent. This was predicated on the assumption that the concentrations of pharmaceuticals in the effluent could potentially pose a risk to the aquatic environment.

The present study has focused on the most frequently detected pharmaceuticals in wastewater and surface water, as reported in the extant literature [8,9,13,22,38,39]. The pharmaceuticals in question are divided into the following groups: β-blockers, antidepressants, non-steroidal anti-inflammatory drugs (NSAIDs), antibiotics and antihistamines. The substances have been selected based on their chemical stability and low degradation rates.

## Materials and methods

### Sampling area and sample collection

**Fig 1:**
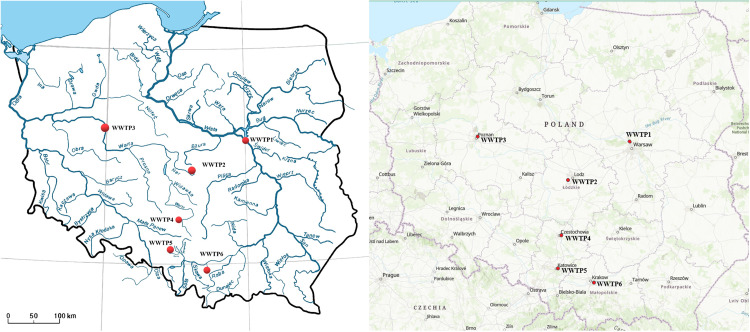
The locations of six WWTPs. (Reprinted from USGS National Map Viewer -public domain, https://apps.nationalmap.gov/viewer/).

**Fig 2:**
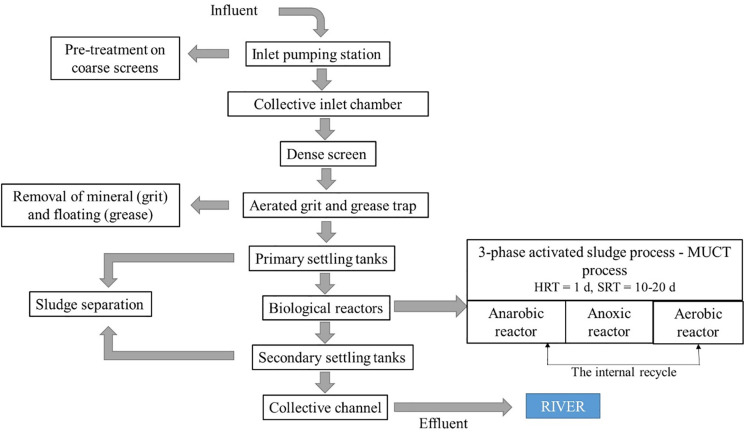
Schematic diagram of the treatment processes in the six WWTPs.

Poland is located within the catchment areas of the Baltic (99% of the territory), Black and North Seas. The main rivers are the Vistula and the Oder, which drain 54% and 28% of the Polish territory respectively. Six wastewater treatment plants (WWTPs) with mechanical-biological treatment technology using conventional activated sludge (CAS) were selected for the study. The selection of WWTPs was based on their location within major urban agglomerations in Poland (Fig 1) with a service population of over 200,000. Notably, all selected WWTPs are situated on the borders of the catchment areas of the two largest rivers in Poland, the Vistula and the Oder. The hydrological network in Poland is illustrated in Fig 1, while the characteristics of the individual WWTPs are presented in Table 1. Fig 2 presents a schematic diagram of the wastewater treatment process at six WWTPs.

**Table 1:** Characteristics of the WWTPs.

WWTPs number	Latitude and longitude	Inhabitants number served by WWTPs	Daily average flow rate Qd^*^ m^3^/d	Daily maximum flow rate Qm^*^ m^3^/d	Daily average sewage sludge production P_d_^*^ Kg/d dw	Type of wastewater	Industrial wastewater contribution %	Wastewater receiver
WWTP1	52°35’10.0”N 20°96’00.2”E	2,100,000	435,300	515,000	94,707	Urban Stormwater Industrial	27%	Vistula
WWTP2	51°72’92.9”N 19°34’50.8”E	1,026,260	180,000	332,000	45,107		12%	Ner, the catchment of Odra
WWTP3	52°43’10.3”N 16°96’02.4”E	350,000	50,000	85,400	8,107		29%	Warta, catchment of Odra
WWTP4	50°81’80.5”N 19°16’44.3”E	313,385	40,136	88,000	6,984		11%	Warta, catchment of Odra
WWTP5	50°26’48.4”N 19°07’14.8”E	200,000	40,000	40,000	6,493		3%	Rawa, catchment of Vistula
WWTP6	50°03’14.0”N 20°01’76.9”E	780,000	165,000	328,000	41,823		4%	Drwina, catchment of Vistula

* Qd, Qm, Pd, are average values from the years in which the research was conducted.

Samples were collected weekly in July for three years using automated samplers. The sampling period lasted 72 hours, with samples taken hourly. This process covered both the influent and effluent streams. The samples were taken in proportion to the flow rates of the influent and effluent, and the results were then averaged. Influent samples were collected from the inlet chamber, while effluent samples were gathered from the collection channels. Sludge samples were collected once a week after dewatering. Samples were collected in plastic containers and transported immediately to the laboratory. Samples were stored at −20°C, protected from light and air, until further analysis. The characteristics of the effluent are presented in Tables A and B in S1 File.

The following types of active pharmaceutical ingredients were selected for the study: antibiotics: Sulfamethoxazole (SUL), non-steroidal anti-inflammatory drugs: Ibuprofen (IBU), Naproxen (NAP), Diclofenac (DIC), Ketoprofen (KET), antihypertensive drugs: Atenolol (ATE), Propranolol (PRO), Matoprolol (MET), Furosemide (FUR) and neuroactive drugs: Carbamazepine (CAR), Mianserin (MIA), Kluoxetine (FLU). Additionally antihistamines drug Loratadine (LOR) and salicylic acid (SAL) were tested. The physicochemical characteristics of the individual pharmaceuticals are shown in the Table C in S1 File.

### Analytical methods

The Oasis HLB and Oasis MCX columns were first conditioned with methanol and water mixture (1:1, v/v). Then, 100 mL of collected wastewater samples were passed through Oasis HLB and Oasis MCX in succession The deposits from the columns were then desiccated under vacuum conditions.The analytes were then extracted using 6 mL of methanol, followed by a mixture of methanol and 0.1% formic acid. The extracts were concentrated under a nitrogen stream. After concentration, the enriched extracts were diluted to a final volume of 1 mL with a methanol-water mixture containing 0.1% formic acid. Finally, the solution was filtered through a syringe filter and analyzed using liquid chromatography-mass spectrometry (LC-MS).

The 0.5 g of collected sludge samples were lyophilised, and placed in Falcon tubes, followed by the addition of acetonitrile: ethyl acetate (1:1, v/v) with 10% acetic acid and shaken vigorously for 1 min. The samples were then extracted at 50 °C for 10 min using ultrasound and centrifuged at 5000 rpm. The solutions of the sediments obtained were decanted. The procedure was repeated twice with a fresh portion of the extraction mixture. The decanted extracts were concentrated in a nitrogen stream. The residues were dissolved in 1 ml of a mixture of MeOH: H_2_O (1:1, v/v) with 0.1% formic acid. The filtered extracts were subjected to chromatographic analysis using the LC-MS method.

Chromatographic analysis was performed on a Shimadzu LC-MS/MS 8050 chromatograph. The chromatographic conditions are presented in Tables D and E in S1 File. Quantitative analysis was performed using the calibration curve method. The R2 coefficient for the calibration curves was between 0.997 and 0.999. The LOD and LOQ are presented in Table F in S1 File.

Analyses were performed in triplicate. The method was validated using fortified samples. The recoveries ranged from 68% to 94% for wastewater and 40% to 70% for sewage sludge. The RSD ranged from 1% to 8% for wastewater and 10% to 25% for sewage sludge. The results of the analysis are calculated in terms of recovery. The RSD and recovery data are presented in Table F in S1 File.

#### Data calculations.

The analysis results of individual pharmaceuticals in wastewater and sewage sludge were normalized based on the average daily wastewater flow, the daily amount of sewage sludge produced, and the number of inhabitants served by the studied wastewater treatment plants. The following equations were used.


$${\text{M}}_{\text{i}}=\frac{{\text{C}}_{\text{i}}\times {\text{Q}}_{\text{d}}}{{\text{10}}^{\text{6}}}$$ (1)

$${\text{M}}_{\text{e}}=\frac{{\text{C}}_{\text{e}}\times {\text{Q}}_{\text{d}}}{{\text{10}}^{\text{6}}}$$ (2)

$${\text{M}}_{\text{ss}}=\frac{{\text{C}}_{\text{s}s}\times {\text{P}}_{\text{d}}}{{\text{10}}^{\text{6}}}$$ (3)

$$\text{RE}\%=\frac{\left({\text{M}}_{\text{i}}-{\text{M}}_{\text{e}}-{\text{M}}_{\text{s}s}\right)}{{\text{M}}_{\text{i}}}\times \text{100}$$ (4)

$${\text{M}}_{\text{load}}/\text{1000 inhabitants }=\frac{{\text{M}}_{\text{i}}\times \text{1000}}{\text{Ni}}\times 10^{3}$$ (5)

$$\text{Emis}/\text{1000 inhabitants }=\frac{\left({\text{M}}_{\text{e}}+{\text{M}}_{\text{ss}}\right)\times \text{1000}}{\text{Ni}}\times 10^{3}$$ (6)

Where Ci and Ce (ng/L) are the concentrations of individual PPCPs in the influent and effluent, respectively, Css (µg/kg) is the content of individual PPCPs in the sludge. Qd (m^3^/d) is the average daily flow and Pd (kg/d) is the daily sludge production in STPs (Table 1). Mi, Me and Mss (g/d) are the daily mass fluxes of PPCPs in influent, effluent and sludge, respectively. RE (%) is the removal efficiency of individual PPCPs during the treatment process in the WWTP. Mload/1000 p.e. (mg/d/1000 p.e.) is the daily load of individual PPCPs in the influent per 1000 p.e. Emis/1000 p.e. (mg/d/1000 p.e.) is the daily emission of individual PPCPs per 1000 p.e. Ni is the number of inhabitants served by the WWTP (Table 1) [10,16,21,39].

## Results and discussion

### Presence of pharmaceuticals in WWTPs

The mean concentrations of the pharmaceuticals under study in the influent, effluent, and sewage sludge collected from sixsewage treatment plants (WWTPs) are presented in Table 2. The results of the pharmaceutical content for each treatment plant, together with the calculated data according to Equations 1–6, can be found in Table G in S1 File. Atenolol, propranolol, and mianserin were not detected in any effluent samples from the examined plants, and atenolol was not detected in the sludge samples. In the influent, the concentrations of pharmaceuticals ranged from 7 ng/L to 1019 ng/L, with the highest concentrations recorded for the following compounds: ketoprofen (ranging from 249 ng/L to 1019 ng/L), sulfamethoxazole (ranging from 89 ng/L to 693 ng/L), and carbamazepine (ranging from 22 ng/L to 624 ng/L). The concentrations of the other pharmaceuticals investigated did not exceed 229 ng/L. Salicylic acid was present only in WWTP2. In the effluent, the concentrations of pharmaceuticals ranged from 9 ng/L to 2266 ng/L, with the highest concentrations detected for the following compounds: fluoxetine (204–2266 ng/L), carbamazepine (303–1555 ng/L), sulfamethoxazole (96–974 ng/L), diclofenac (100–851 ng/L) and ibuprofen (89–645 ng/L). Concentrations of other drugs did not exceed 417 ng/L. Loratadine was detected only in the samples from WWTP3. In sewage sludge, the levels of pharmaceuticals ranged from 1.76 μg/kg dw to 406 μg/kg dw, with the highest levels detected for the compounds: fluoxetine (164–406 μg kg^-1^ dw), carbamazepine (13–119 μg/kg dw) and metoprolol (8.5–28 μg/kg dw). Mianserin was detected only in WWTP6 (161 μg/kg dw), while propranolol was detected in WWTP4 (92 μg/kg dw) and sulfamethoxazole in WWTP5 (1.76 μg/kg dw). The presence of the other pharmaceuticals was not detected in the sewage sludge.

**Table 2:** The min-max, mean concentration, frequency of detection and removal efficiency of pharmaceuticals in influent, effluent and sewage sludge from 6 WWTPs.

Name of substance	influent			effluent			sewage sludge			RE range min-max %
	mean (n = 6) ng/L	range min-max ng/L	freq %	mean (n = 6) ng/L	range min-max ng/L	freq %	mean (n = 6) µg/Kg dw	range min-max µg/Kg dw	freq %	
IBU	23	18-74	50	208	18-654	67	nd	–	–	N-100
NAP	54	17-229	50	nd	–	–	nd	–	–	91-100
DIC	7	3-14	33	292	100-851	100	nd	–	–	N
KET	668	249-1019	100	55	2-171	83	nd	–	–	66-100
SUL	279	89-693	100	324	2.4-974	83	0.9	1-1.76	17	N-100
ATE	nd	–	–	nd	–	–	nd	–	–	–
PRO	nd	–	–	nd	–	–	16	4-92	17	N
MET	103	2-191	83	199	2-417	83	20	8.5-28	100	N
FUR	87	2.1-180	83	108	2-495	80	nd	–	–	N-100
CAR	265	22-624	100	792	303-1555	100	46	13-119	83	N
MIA	nd	–	–	nd	–	–	27	4-161	17	N
FLU	68	4-212	67	787	204-2266	100	301	164-406	83	N
LOR	nd	–	–	40	2-223	17	nd	–	–	N
SAL	34	2.1-190	17	nd	–	–	nd	–	–	98-100

Freq – Frequency of detection, RE – removal efficiency, N – negative value, nd – not detected.

The findings revealed that the concentration of pharmaceuticals in the influent, effluent, and sludge did not depend on the number of people served by the investigated WWTPs. This observation contradicts the results obtained in other studies [21,40]. As shown in Table 2, the maximum concentrations of pharmaceuticals in the influent and effluent of WWTPs in Poland did not exceed the maximum levels found in other global studies, and in many cases, the mean results were comparable (see Table 3). For instance, the mean concentrations of ibuprofen, naproxen, diclofenac, ketoprofen, sulfamethoxazole and salicylic acid in influent and effluent samples collected from WWTPs in Ireland, Spain, Canada and Algiers were higher than the maximum values obtained in this study. However, the mean concentrations of the studied pharmaceuticals in Poland were at a similar level to those in Italy, Korea or China [16,18,27,30–32] In comparison to a European study of 90 WWTPs [33], which examined the mean concentration of select pharmaceuticals (see Table 3), the values obtained in this study were much higher for ibuprofen and diclofenac, while the concentration of ketoprofen was similar (see Table 2). The mean concentrations of sulfamethoxazole and carbamazepine were similar to those detected in the Polish WWTPs, while the mean concentration of fluoxetine was much higher. The concentration of pharmaceuticals in the previous study in Poland (Table 3) did not deviate from the concentration presented in this study (Table 2).

**Table 3:** The min-max, mean concentrations of pharmaceuticals in influent, effluent and sewage sludge, as reported in the literature.

Name of substance	influent range in Poland ^a^ ng/L	effluent range in Poland^a^ ng/L		influent worldwide range ^c^ ng/L	effluent worldwide range ^c^ ng/L	sewage sludge worldwide range ^d^ µg/kg dw
			effluent mean content in 90 European WWTPs^b^			
IBU	280-34508	19.5-110	80.5	300-14600	44-2129	0.5-90
NAP	240-22247	28-70	26.7	9.2-9584.8	0.71-958	
DIC	460-4477	120-5630	49.5	49-2318.5	14-2710.7	0.8-14
KET	79.4-233.6	87.4-257	86.0	4.5-668	1.4-1653	70-100
SUL			280	25-2500	9.1-1000	0.1-2.9
ATE					0.3-111	
PRO				1.2-75	2-310	0.05-29.6
MET				59-1700	184-4340	0.01-226
FUR				450-6450	14-2280	
CAR			832	3.9-550	6.4-4609	1.80-57
MIA					1-62.3	
FLU			2.1		5-21.5	50-100
LOR						
SAL	40.3-1400	12.1-470		12800-38100		

^a^ [26,37], ^b^ [33] ^c^ [5,16,18,27,29–32], ^d^ [16,21,30].

The results presented in this paper on pharmaceutical concentrations in effluent were compared with data from 90 European WWTPs [33]. According to Loss et al., the compounds most frequently detected in the examined effluents were carbamazepine, diclofenac and sulfamethoxazole, with detection frequencies of 90%, 89% and 83%, respectively. In tests carried out in Poland, carbamazepine and diclofenac were detected in all cases, and sulfamethoxazole in 83% of cases. Carbamazepine and sulfamethoxazole were also detected in sewage sludge, with detection frequencies of 83% and 17%, respectively. Loss et al. found naproxen, ibuprofen and ketoprofen with frequencies of detection of 66%, 57% and 48%, respectively. The detection frequency of mianserin in effluent was no more than 28%, while the detection frequency of fluoxetine was 22%. In contrast to the findings of this study, naproxen was not detected in the effluent of any of the WWTPs examined. Ibuprofen and ketoprofen, however, were present in 67% and 83% of the samples, respectively. Mianserin was not detected in the effluent of any of the WWTPs included in this study, whereas fluoxetine was detected in all effluent samples.

### The removal efficiency of pharmaceuticals in WWTPs

The mass loads of the pharmaceuticals under investigation were calculated according to Equations 1–3. The removal efficiencies of the pharmaceuticals were calculated using Equation 4. The removal efficiencies of individual pharmaceuticals in the WWTPs under study are presented in Table 2.

In the context of wastewater treatment processes, the presence of naproxen and salicylic acid was observed to be completely eliminated in all the WWTPs that were the subject of this study. A similar trend was noted with ketoprofen, which was also effectively removed, with removal efficiencies ranging from 66% (WWTP6) to 100% (WWTP5). Ibuprofen, on the other hand, exhibited a removal efficiency of up to 86% in WWTP5, while in the other investigated WWTPs, this compound was found to exhibit negative removal rates. The removal efficiency of sulfamethoxazole was 100% in WWTP3; however, in WWTP5 and WWTP6, it was only 0.5% and 1%, respectively, and in most cases, the sulfamethoxazole removal efficiency displayed negative values. The removal efficiency of furosemide was 98% in WWTP3 and 96% in WWTP5. In contrast, the removal efficiency of furosemide was 30% in WWTP1, while in the other WWTPs investigated, the removal efficiency showed a negative value. Diclofenac, carbamazepine, and fluoxetine were not effectively removed in any of the investigated wastewater treatment plants, resulting in negative removal values. Metoprolol was not effectively removed, demonstrating negative removal efficiency in all the wastewater treatment plants (WWTPs) studied. Mianserin, loratadine, and propranolol also exhibited negative removal efficiencies, with mianserin and propranolol being detected solely in sewage sludge.

The removal efficiency of the studied pharmaceuticals varied significantly, ranging from complete removal to none, depending on the specific compound and the wastewater treatment plant [1,22,33]. For example, the diclofenac compound is reported to be generally poorly removable during wastewater treatment, with removal values often reported as negative [4,18,22,26,39]. Many studies have shown that wastewater treatment systems based on the conventional activated sludge (CAS) method do not effectively remove compounds such as diclofenac, carbamazepine or sulfamethoxazole, and their removal efficiencies are often negative [4,5,18,19,30,39]. Removal efficiencies for ibuprofen are primarily reported to be between 70% and 100% [3,5,18,22,39]. However, some studies have also reported negative removal efficiencies for this compound [22]. The reported removal efficiencies of fluoxetine and furosemide range from 20% to 100% [22]. The available literature on loratadine indicates that its removal rates do not exceed 40% [22]. Loratadine is an antihistamine that is typically found in wastewater, mostly during the spring and early summer [34]. This seasonal occurrence may explain why loratadine was detected in only one of the wastewater treatment plants (WWTPs) examined in this study. The presence of this compound in one or two investigated sites has also been reported in other studies [40].

The negative removal efficiency indicates that the concentration of pharmaceuticals in the effluent was higher than in the influent. Most pharmaceuticals enter wastewater through faeces and urine, existing as a mixture of parent compounds and glucuronic acid conjugates. During the biological wastewater treatment process, these conjugates may revert to their parent compounds, potentially increasing the concentration of pharmaceuticals in the wastewater [33]. It has been reported that the occurrence of diclofenac and its glucuronide in influent samples results in the deconjugation of diclofenac acyl glucuronides back to diclofenac during the biological wastewater treatment process, thereby increasing the concentration of diclofenac in the effluent [33,41]. Furthermore, pharmaceuticals have been observed to become entrapped within faecal particles in wastewater, with a subsequent gradual release during the wastewater treatment process. This phenomenon can lead to an enhancement in the concentrations of pharmaceuticals in the effluent [33].

All the WWTPs examined in the study employed the same type of wastewater treatment process. However, their efficiency in removing the same pharmaceuticals varied. Similar patterns have been observed in wastewater treatment plants around the world, where the removal efficiency of individual pharmaceuticals differs across various WWTPs, even when the same treatment parameters are applied [18,39]. The effectiveness of biological treatment for organic compounds in wastewater relies on several factors, including oxygen levels, temperature, and the type and quantity of bacteria present. This explains the significant variations in treatment efficiency observed in different WWTPs that use the activated sludge method (CAS). Hydraulic retention time (HRT) and solids retention time (SRT) in conventional activated sludge (CAS) systems has been a subject of considerable research [1]. The prevailing HRT values in the examined WWTPs were found to be approximately one day, with a typical range of 20–40 hours. The variation in HRT values among the WWTPs was minimal, totalling less than 12 hours, suggesting that this variation does not have a significant impact on removal efficiency. The estimated SRT ranged from 10 to 20 days. However, WWTPs serving populations of over 200,000 do not typically operate under steady conditions due to daily fluctuations in the influent load to the bioreactors, thus hindering the assessment of the effect of SRT on removal efficiency.

### Emission of pharmaceuticals to the environment

**Fig 3:**
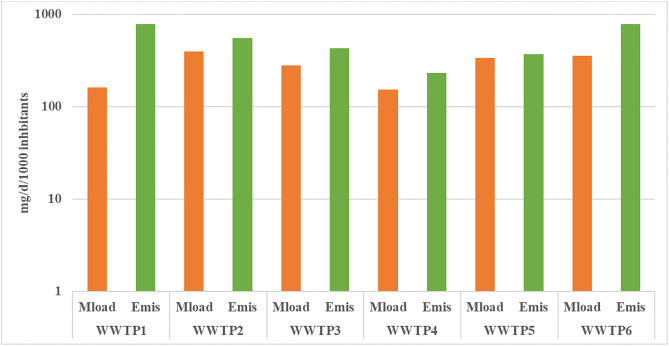
Daily mass load (Mload) and daily emission (Emis) of sum pharmaceuticals per 1000 inhabitants.

To normalise the results and enable comparison between treatment plants of different sizes, the results are divided by the population served by the plant in question. The mass load typically equates to 1,000 inhabitants and were calculated using equations 5 and 6, with the results displayed in Fig 3. WWTP2, WWTP5 and WWTP6 exhibited the highest values (337–397 mg/d/1000 inhabitants) of the mass load per 1000 inhabitants. Furthermore, WWTP1 and WWTP6 exhibited the highest discharges of pharmaceuticals to the environment (785 mg/d/1000 inhabitants and 778 mg/d/1000 inhabitants, respectively). For the remaining WWTPs, the mass load of pharmaceuticals ranged from 153 mg/d/1000 inhabitants (WWTP1) to 279 mg/d/1000 inhabitants (WWTP5). The range of emissions to the environment was from 231 mg/d/1000 inhabitants (WWTP5) to 552 mg/d/1000 inhabitants (WWTP4). It was found that the daily emission of pharmaceutical compounds to the environment exceeded the daily mass load in all WWTPs investigated.

The average daily discharge of pharmaceuticals from 6 WWTPs was 524 mg/d/1000 inhabitants. This corresponds to 191 g of pharmaceuticals per 1000 inhabitants per year. Given that each of the agglomerations under study has a minimum population of 200,000 inhabitants, the annual emission of pharmaceuticals into rivers is a minimum of 40 mg.

**Fig 4:**
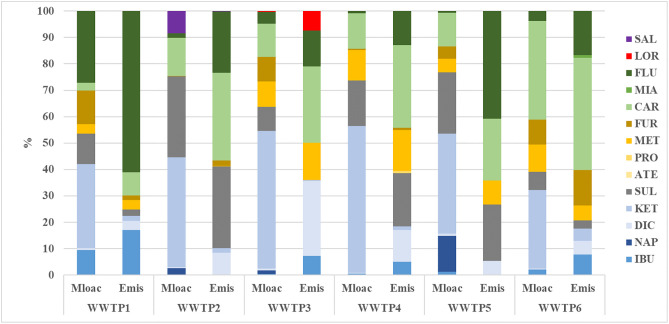
Percentage of individual pharmaceuticals in daily load (Mload) and daily emission (Emis) per 1000 inhabitants.

The percentages of individual pharmaceuticals in the daily mass load and emissions are shown in Fig 4. In each of the investigated WWTPs, ketoprofen accounted for the highest rate of the daily mass load/1000 inhabitants (29–55%), followed by sulfamethoxazole (7–30%), carbamazepine (3–37%) and fluoxetine (1–27%). In WWTP1, WWTP3 and WWTP6, the compound furosemide was also present in significant proportions (9–13%). The above mentioned compounds are listed as the most commonly occurring compounds in the influent [22]. The percentage of individual compounds in the total amount of pharmaceuticals discharged to the environment was not always proportional to the number of pharmaceuticals entering the wastewater treatment plant. The percentage of pharmaceuticals discharged to the environment also varied considerably between WWTPs. In WWTP1, fluoxetine and ibuprofen accounted for the highest percentage of pharmaceuticals discharged to the environment (61% and 17%, respectively), in WWTP2: carbamazepine (34%), sulfamethoxazole (32%) and fluoxetine (21%), in WWTP3: carbamazepine (29%), diclofenac (28%) and metoprolol (14%), in WWTP4: carbamazepine (32%), sulfamethoxazole (21%) and diclofenac (12%), in WWTP5: carbamazepine (24%) and metoprolol (12%): carbamazepine (32%), sulfamethoxazole (21%) and diclofenac (12%), in WWTP5: carbamazepine (24%) and sulfamethoxazole (22%), and in WWTP6: carbamazepine (43%), fluoxetine (15%) and furosemide (14%). Except for fluoxetine and furosemide (insufficient data available), all compounds are listed as the most frequently emitted to the environment [1,18,20,22,39].

### Risk assessment of pharmaceuticals in aquatic species

The potential ecological risk of individual pharmaceuticals was assessed based on the risk quotient (RQ), which was calculated as the ratio between the maximum measured concentration (MEC) of a given pharmaceutical in wastewater (ng/L) and the predicted no-effect concentration (PNEC) (ng/L) [23,36,41,42]. The PNECs used for the risk assessment are shown in Table 3 and represent the lowest ecotoxicological PNECs reported in the literature for three groups of aquatic organisms: algae, daphnia and fish. The risk ranking criteria employed in this study were as follows: RQ < 0.1 – minimal risk, 0.1 ≤ RQ < 1 – moderate risk, RQ ≥ 1 – high risk [23,31,40,43]. The maximal RQ values for the pharmaceuticals studied are shown in Table 4. The RQ values for each WWTPs are shown in the Table H in S1 File.

**Table 4:** Environmental risk assessment (RQ) of the maximum concentration of pharmaceuticals in effluent.

Name of substances	Algae	Daphnia	Fish	PNEC µg/L
IBU	*0.161*	0.072	*0.129*	Algae 4.00 ^a,b^ Daphnids 9.02 ^a,b^ Fish 5.00 ^a,b^
NAP	nd*	nd*	nd*	Algae 22.0 ^a,b^ Daphnids 15.0 ^a,b^ Fish 34.0 ^a,b^
DIC	0.059	0.039	0.002	Algae 14.5 ^a,b^ Daphnids 22.0 ^a,b^ Fish 532 a,b
KET	**1.425**	0.007	0.000	Algae 164 ^a,b^ Daphnids 248 ^a,b^ Fish 32.0 ^a,b^
SUL	0.012	0.012	0.001	Algae 0.120 ^a^ Daphnids 25.2 ^a,b^ Fish 562 ^a,b^
ATE	nd*	nd*	nd*	Algae 78.0 ^a^ Daphnids 83.0 ^a^ Fish 1500 ^a^
PRO	nd*	nd*	nd*	Algae 5.50 ^a^ Daphnids 2.30 ^a^ Fish 30.0 ^a^
MET	0.003	0.007	0.001	Algae 7.9 ^b^ Daphnids 63.9 ^b^ Fish 944 ^b^
FUR	0.003	0.008	0.001	Algae 142 ^c^ Daphnids 60.62 ^d^ Fish 497 ^c^
CAR	0.018	0.020	0.044	Algae 85.0 ^a,b^ Daphnids 76.3 ^a,b^ Fish 35.4 ^a,b^
MIA	–	–	–	nd ^e^
FLU	**2.833**	**4.443**	**1.333**	Algae 0.800 ^a,b^ Daphnids 0.510 ^a,b^ Fish 1.70 ^a,b^
LOR	**4.551**	**1.570**	**10.619**	Algae 0.049 ^f^ Daphnids 0.142^f^ Fish 0.021^f^
SAL	–	–	–	nd ^e^

PNEC predicted no-effect concentration, * nd – not detected.

^a^ [42] ^b^ [44], ^c^ [43], ^d^ [45], ^e^ No data (no toxicity data was found), ^f^ Indicates predicted (Q)SAR values [40].

As demonstrated in Table 4, the findings of this study indicate that fluoxetine and loratadine pose a moderate to high risk to all three groups of aquatic organisms. The PNEC values for loratadine were derived through the calculation of predicted (Q)SARs, which necessitate careful consideration due to their potential endocrine-disrupting effects. These effects are not incorporated into (Q)SAR calculations and consequently, the potential risk may differ considerably from the estimated risk [40]. Fluoxetine and ibuprofen have been frequently reported in the literature as posing a high risk to aquatic organisms [22,42,46], however, in this study, ibuprofen barely showed a moderate risk (Table 3). Diclofenac and naproxen have also been identified as posing a high risk [15,43,46,47]. However, these pharmaceuticals exhibited a low risk in the present study. Ketoprofen was found to pose a moderate to high risk to algae and a low risk to daphnia and fish. While ketoprofen is generally classified as low risk in the literature [22,43], studies conducted in wastewater and rivers have indicated a high risk [3]. The remaining pharmaceuticals tested exhibited minimal risk to aquatic organisms.The presence of pharmaceutical active substances in treated wastewater has been identified as posing the greatest risk to the aquatic environment. This is due to their chemical stability and slow degradation process. Furthermore, the presence of retained pharmaceutical active substances in sewage sludge may pose a risk if they are incorporated into the food chain during natural utilisation. The findings of this study will lay the groundwork for research into the inactivation of pharmaceutical active substances and their metabolites in sewage and sewage sludge.

## Conclusions

The study’s findings demonstrated that municipal wastewater treatment facilities are a source of pharmaceuticals being released into the environment. Conventional mechanical-biological treatment processes (CAS), have been found to be ineffective at removing these pharmaceuticals from wastewater. Discharging pharmaceuticals into the aquatic environment via wastewater poses a significant threat to aquatic organisms. The investigation found that influent samples had the highest concentrations of sulfamethoxazole, ketoprofen and carbamazepine.The effluent samples showed the highest levels of fluoxetine, carbamazepine and sulfamethoxazole..The highest concentrations of carbamazepine, fluoxetine and metoprolol were found in sewage sludge.

During the wastewater treatment processes, naproxen and salicylic acid were the only compounds effectively removed. The removal efficiencies of ketoprofen ranged from 66 to 99%, while the removal efficiencies of ibuprofen, sulfamethoxazole and furosemide ranged from negative values to 99%. During the CAS wastewater treatment process, diclofenac, metoprolol, propranolol, carbamazepine, fluoxetine, loratadine and mianserin were not removed from the influent (negative removal efficiency).

Daily emissions of pharmaceuticals to the environment exceeded the daily mass load. The annual emissions of pharmaceuticals to rivers from wastewater treatment plants in the study area amounted to at least 40 Mg. Ketoprofen, sulfamethoxazole, carbamazepine and fluoxetine were identified as the primary contributors to the total mass load and emissions of pharmaceuticals, at up to 56%, 31%, 38% and 27% respectively.

Fluoxetine and loratadine posed the highest risk to the three groups of aquatic organisms tested (algae, Daphnia and fish), while ibuprofen posed a moderate risk. Sulfamethoxazole posed a high risk only to algae. The other pharmaceuticals did not pose a risk to the selected aquatic organisms.

## Supporting information

S1 File Detailed information on the methodology and the results of the research. (DOCX)
