# Supplementary material for: Removal efficiency of pharmaceuticals during the wastewater treatment process: Emission and environmental risk assessment
Source: PLoS One. 2025 Sep 24;20(9):e0331211. doi: 10.1371/journal.pone.0331211 (PMC12459797; doi:10.1371/journal.pone.0331211)
Supplement: S1 File — (DOCX) [file pone.0331211.s001.docx]

**Supporting Information**

**REMOVAL EFFICIENCY OF PHARMACEUTICALS DURING THE WASTEWATER TREATMENT PROCESS. EMISSION AND ENVIRONMENTAL RISK ASSESSMENT**

**Paulina Chaber-Jarlachowicz^1*¶^, Barbara Gworek^1¶^, Radosław Kalinowski^2&^**

^1^Department of Environmental Chemistry and Risk Assessment, Institute of Environmental Protection – National Research Institute, Warsaw, Poland

^2^RADIKAL Science Consultancy, Warsaw, Poland

*Corresponding author: Paulina Chaber-Jarlachowicz

Email: [paulina.chaber-jarlachowicz@ios.edu.pl](mailto:paulina.chaber-jarlachowicz@ios.edu.pl)

^¶^These authors contributed equally to this work.

^&^These authors also contributed equally to this work.

**Table A. Influence characteristic**

|  | WWTP1 W | WWTP2 L | WWTP3-P | WWTP4-Cz | WWTP5-Kt | WWTP6-Kr |
| --- | --- | --- | --- | --- | --- | --- |
| BOD  mgO_2_/L | 254 | 358 | 383 | 293.8 | 355 | 408 |
| COD  mgO_2_/L | 573 | 866 | 1035 | 628.3 | 886 | 700 |
| TSSC*  mg/L | 314 427 182 | 405 | 477 | 371.4 | 372 | 314 |
| Total nitrogen  mg/L | 51.92 | 55 | 82 | 77.0 | 83 | 58 |
| Total phosphorus  mg/L | 5.99 | 7 | 11 | 8.0 | 10 | 6 |

* Total suspended solid content

**Table B. Effluent characteristic**

|  | WWTP1 | WWTP2 | WWTP3 | WWTP4 | WWTP5 | WWTP6 |
| --- | --- | --- | --- | --- | --- | --- |
| BOD  mgO_2_/L | 4.7 18 33- | 8 | 4 | 4.0 | 2 | 5 |
| COD  mgO_2_/L | 28.7 92 61 | 47 | 39 | 34.5 | 32 | 29 |
| TSSC*  mg/L | 7.5 18 19 | 13 | 6 | 6.3 | 4 | 6 |
| Total nitrogen  mg/L | 7.66 | 10 | 5 | 8.2 | 8 | 8 |
| Total phosphorus  mg/L | 0.29 | 1 | 0.25 | 0.28 | 1 | 0.3 |

* Total suspended solid content

**Table C Chemical structure, basic properties and** **predicted no-effect concentration (PNEC) of pharmaceuticals**

|  | Name of substances | Abbreviation | Molecular weight  g/mol | Molecular formula | log Kow | Solubility in water at 25°C  Mg/L |
| --- | --- | --- | --- | --- | --- | --- |
| non-steroidal anti-inflammatory drugs (NSAIDs) | Ibuprofen  15687-27-1 | IBU | 206.29 | C_13_H_18_O_2_ | 3.72 | 21 |
|  | Naproxen  22204-53-1 | NAP | 230.26 | C_14_H_14_O_3_ | 3.00 | 15.9 |
|  | Diclofenac  15307-86-5 | DIC | 295.02 | C_14_H_11_Cl_2_NO_2_ | 4.06 | 2.37 |
|  | Ketoprofen  22071-15-4 | KET | 254.28 | C_14_H_14_O_3_ | 3.12 | 51 |
| Antibiotics | Sulfamethoxazole  723-46-6 | SUL | 253.28 | C_10_H_11_N_3_O_3_S | 0.89 | 610 |
| Antihypertensive drugs | Atenolol  29122-68-7 | ATE | 266.34 | C_20_H_24_O_2_ | 0.097 | 13300 |
|  | Propranolol  525-66-6 | PRO | 259.35 | C_16_H_21_NO_2_ | 3.10 | 61.7 |
|  | Metoprolol  37350-58-6 | MET | 267.36 | C_15_H_25_NO_3_ | 1.79 | 16900 |
|  | Furosemide  54-31-9 | FUR | 330.75 | C_12_H_11_ClN_2_O_5_S | 3.00 | 73.1 |
| Neuroactive drugs | Carbamazepine  298-46-4 | CAR | 236.27 | C_15_H_12_N_2_O | 2.67 | 17.7 |
|  | Mianserin  24219-97-4 | MIA | 264.37 | C_18_H_20_N_2_ | 4.24 | 0,000232 |
|  | Fluoxetine  54910-89-3 | FLU | 309.3 | C_17_H_18_F_3_NO | 4.09 | 0.05 |
| Antihistamines | Loratadine  79794-75-5 | LOR | 385.88 | C_22_H_23_ClN_2_O_2_ | 5.20 | 0.011 |
| Others | Salicylic acid  69-72-7 | SAL | 138.12 | C_7_H_6_O_3_ | 2.97 | 2240 |

**Table D. Chromatographic conditions**:

| Apparatus | Shimadzu LC-MS/MS 8050 |
| --- | --- |
| HPLC column | Kinetex 2.6 μm C18 column (100x4.6 mm) |
| Detector | MS/MS operating in the MRM mode |
| Oven temperature | 30°C |
| Eluent A | MeOH |
| Eluent B | 1% HCOOH in H2O |
| Eluent (gradient) | 0.01 min - 10% MeOH, 3.00 min - 95% MeOH, up to 7 min, 7 min - 10% MeOH, up to 10 min |
| Flow rate | 0.5 mL/min |
| Volume of injection | 10 µL |

**Table E. The MMR transitions of pharmaceuticals**

| Compound | t_R_  min | ESI | Precursor Ion | Product Ion | CE(eV) |
| --- | --- | --- | --- | --- | --- |
| IBU | 5.209 | - | 205.20 | 161.00 | 35 |
| NAP | 4.886 | - | 229.15 | 169.10 | 35 |
| DIC | 5.152 | - | 294.05 | 178.15 | 35 |
| KET | 4.797 | + | 255.10 | 105.05 | -35 |
| SUL | 3.733 | + | 254.00 | 108.10 | -35 |
| ATE | 2.819 | + | 267.00 | 145.10 | -35 |
| PRO | 4.026 | + | 260.15 | 56.15 | -35 |
| MET | 3.630 | + | 268.15 | 103.10 | -35 |
| FUR | 4.245 | - | 329.05 | 126.00 | 35 |
| CAR | 4.568 | + | 237.08 | 193.10 | -35 |
| MIA | 4.029 | + | 265.00 | 193.10 | -35 |
| FLU | 4.296 | + | 310.05 | 117.10 | -35 |
| LOR | 5.184 | + | 383.10 | 267.15 | -35 |
| SAL | 4.492 | - | 137.10 | 92.90 | 35 |

**Table F. LOD, LOQ, precision and accuracy results in wastewater and sewage sludge samples**

| compound | wastewater | | | | sewage sludge | | | |
| --- | --- | --- | --- | --- | --- | --- | --- | --- |
|  | LOD^1^  ng/L | LOQ^2^  ng/L | %RSD  n=6  (3 ng/L)* | %recovery  n=6  (3 ng/L)* | LOD^1^  µg/kg dw | LOQ^2^  µg/kg dw | %RSD  n=6  7 µg/kg dw* | %recovery  n=6  7 µg/kg dw* |
| IBU | 1.8 | 2.2 | 3.4 | 88 | 1 | 4 | 13 | 80 |
| NAP | 1.7 | 2.1 | 8.3 | 68 | 1 | 3 | 11 | 66 |
| DIC | 1.7 | 2.0 | 3.9 | 76 | 1 | 3 | 15 | 74 |
| KET | 1.7 | 2.0 | 4.8 | 84 | 1 | 3 | 18 | 79 |
| SUL | 2.0 | 2.4 | 2.8 | 71 | 1 | 4 | 9.6 | 72 |
| ATE | 1.6 | 2.0 | 2.5 | 81 | 1 | 3 | 18 | 78 |
| PRO | 1.9 | 2.3 | 4.3 | 92 | 1 | 4 | 12 | 77 |
| MET | 1.6 | 2.0 | 5.9 | 68 | 1 | 3 | 11 | 75 |
| FUR | 1.7 | 2.1 | 1.4 | 72 | 1 | 3 | 10 | 70 |
| CAR | 1.7 | 2.0 | 4.8 | 89 | 1 | 3 | 9.5 | 82 |
| MIA | 2.0 | 2.5 | 3.3 | 90 | 1 | 4 | 12 | 80 |
| FLU | 3.3 | 4.0 | 5.3 | 76 | 2 | 8 | 5.3 | 76 |
| LOR | 1.6 | 2.0 | 2.1 | 94 | 1 | 3 | 11 | 77 |
| SAL | 1.7 | 2.1 | 4.9 | 71 | 1 | 3 | 9.7 | 71 |

*Matrix spike level

^1)^ LOD was set at the lowest level detected by LC/MS/MS.

^2)^ LOQ was set at the lowest calibration level.

**Table G.** **Mean concentrations, daily mass fluxes, removal efficiencies, mass loads and emissions of pharmaceuticals in influent, effluent and sewage sludge for individual WWTPs.**

| **Name of substance** | **influent** | | | **effluent** | | | **sewage sludge** | | | **RE**  **%** | **Mload**  **mg/d/1000 inhabitants** | **Emis**  **mg/d/1000 inhabitants** |  |
| --- | --- | --- | --- | --- | --- | --- | --- | --- | --- | --- | --- | --- | --- |
|  | **mean**  **(n=12)**  **ng/L** | **±SD** | **Mi**  **g/d** | **mean**  **(n=12)**  **ng/L** | **±SD** | **Me**  **g/d** | **mean**  **(n=12)**  **µg/Kg dw** | **±SD** | **Mss**  **g/d** |  |  |  |  |
| **WWTP1** | | | | | | | | | | | | | |
| **IBU** | 74 | 15 | 32.21 | 645 | 84 | 280.8 | < LOD | - | - | N | 15.34 | 133.70 |  |
| **NAP** | < LOD | - | - | < LOD | - | - | < LOD | - | - | - | - | - |  |
| **DIC** | 5 | 3 | 2.18 | 132 | 35 | 57.46 | < LOD | - | - | N | 1.04 | 27.36 |  |
| **KET** | 249 | 38 | 108.4 | 70 | 7 | 30.47 | < LOD | - | - | 72 | 51.61 | 14.51 |  |
| **SUL** | 89 | 6 | 38.74 | 96 | 3 | 41.79 | < LOD | - | - | N | 18.45 | 19.90 |  |
| **ATE** | < LOD | - | - | < LOD | - | - | < LOD | - | - | - | - | - |  |
| **PRO** | < LOD | - | - | < LOD | - | - | < LOD | - | - | - | - | - |  |
| **MET** | 29 | 4 | 12.62 | 126 | 11 | 54.85 | 28 | 4 | 2.65 | N | 6.01 | 27.38 |  |
| **FUR** | 99 | 13 | 43.09 | 69 | 11 | 30.04 | < LOD | - | - | 30 | 20.52 | 14.30 |  |
| **CAR** | 22 | 8 | 9.58 | 303 | 49 | 131.9 | 119 | 7 | 11.27 | N | 4.56 | 68.17 |  |
| **MIA** | < LOD | - | - | < LOD | - | - | < LOD | - | - | - | - | - |  |
| **FLU** | 212 | 53 | 92.28 | 2266 | 141 | 986.4 | 219 | 85 | 20.74 | N | 43.94 | 479.59 |  |
| **LOR** | < LOD | - | - | < LOD | - | - | < LOD | - | - | - | - | - |  |
| **SAL** | < LOD | - | - | < LOD | - | - | < LOD | - | - | - | - | - |  |
| **WWTP2** | | | | | | | | | | | | | |
| **IBU** | < LOD | - | - | < LOD | - | - | < LOD | - | - | - | - | - |  |
| **NAP** | 60 | 14 | 10.80 | < LOD | - | - | < LOD | - | - | 100 | 10.52 | - |  |
| **DIC** | 6 | 3.9 | 1.08 | 265 | 42 | 47.70 | < LOD | - | - | N | 1.05 | 46.48 |  |
| **KET** | 944 | 316 | 169.9 | 55 | 4 | 9.90 | < LOD | - | - | 94 | 165.6 | 9.65 |  |
| **SUL** | 693 | 149 | 124.7 | 974 | 35 | 175.3 | < LOD | - | - | N | 121.5 | 170.8 |  |
| **ATE** | < LOD | - | - | < LOD | - | - | < LOD | - | - | - | - | - |  |
| **PRO** | < LOD | - | - | < LOD | - | - | < LOD | - | - | - | - | - |  |
| **MET** | < LOD | - | - | < LOD | - | - | 24 | 2 | 1.08 | - | - | 1.05 |  |
| **FUR** | 2 | 1 | 0.36 | 64 | 9 | 11.52 | < LOD | - | - | N | 0.35 | 11.23 |  |
| **CAR** | 325 | 107 | 58.50 | 1040 | 39 | 187.2 | 33 | 4 | 1.49 | N | 57.00 | 183.9 |  |
| **MIA** | < LOD | - | - | < LOD | - | - | < LOD | - | - | - | - | - |  |
| **FLU** | 4 | 3 | 0.72 | 651 | 93 | 117.2 | 323 | 39 | 14.57 | N | 0.70 | 128.4 |  |
| **LOR** | < LOD | - | - | < LOD | - | - | < LOD | - | - | - | - | - |  |
| **SAL** | 190 | 61 | 34.20 | < LOD | - | - | < LOD | - | - | 100 | 33.32 | - |  |
| **WWTP3** | | | | | | | | | | | | | |
| **IBU** | 5 | 3 | 0.25 | 216 | 80 | 10.80 | < LOD | - | - | N | 0.71 | 30.86 |  |
| **NAP** | 27 | 5 | 1.35 | < LOD | - | - | < LOD | - | - | 100 | 3.86 | - |  |
| **DIC** | 14 | 3 | 0.70 | 851 | 132 | 42.55 | < LOD | - | - | N | 2.00 | 121.6 |  |
| **KET** | 1019 | 30 | 50.95 | 9 | 3 | 0.45 | < LOD | - | - | 99 | 145.6 | 1.29 |  |
| **SUL** | 177 | 7 | 8.85 | < LOD | - | - | < LOD | - | - | 100 | 25.29 | - |  |
| **ATE** | < LOD | - | - | < LOD | - | - | < LOD | - | - | - | - | - |  |
| **PRO** | < LOD | - | - | < LOD | - | - | < LOD | - | - | - | - | - |  |
| **MET** | 191 | 22 | 9.55 | 417 | 31 | 20.85 | 22 | 2 | 0.18 | N | 27.29 | 60.08 |  |
| **FUR** | 180 | 42 | 9.00 | < LOD | - | - | < LOD | - | - | 100 | 25.71 | - |  |
| **CAR** | 244 | 40 | 12.20 | 865 | 31 | 43.25 | 13 | 1 | 0.11 | N | 34.86 | 123.9 |  |
| **MIA** | < LOD | - | - | < LOD | - | - | < LOD | - | - | - | - | - |  |
| **FLU** | 90 | 36 | 4.50 | 406 | 109 | 20.30 | < LOD | - | - | N | 12.86 | 58.00 |  |
| **LOR** | 2 | 1 | 0.10 | 223 | 39 | 11.15 | < LOD | - | - | N | 0.29 | 31.86 |  |
| **SAL** | < LOD | - | - | < LOD | - | - | < LOD | - | - | - | - | - |  |
| **WWTP4** | | | | | | | | | | | | | |
| **IBU** | 2 | 1 | 0.08 | 89 | 15 | 3.57 | < LOD | - | - | N | 0.26 | 11.40 |  |
| **NAP** | < LOD | - | - | < LOD | - | - | < LOD | - | - | - | - | - |  |
| **DIC** | 6 | 4 | 0.24 | 219 | 33 | 8.79 | < LOD | - | - | N | 0.77 | 28.05 |  |
| **KET** | 661 | 71 | 26.53 | 23 | 6 | 0.92 | < LOD | - | - | 97 | 84.66 | 2.95 |  |
| **SUL** | 206 | 17 | 8.27 | 363 | 23 | 14.57 | < LOD | - | - | N | 26.38 | 46.49 |  |
| **ATE** | < LOD | - | - | < LOD | - | - | < LOD | - | - | - | - | - |  |
| **PRO** | < LOD | - | - | < LOD | - | - | 92 | 3 | 0.64 | N | - | 2.05 |  |
| **MET** | 137 | 11 | 5.50 | 275 | 20 | 11.04 | 19 | 7 | 0.13 | N | 17.55 | 35.64 |  |
| **FUR** | 7 | 1 | 0.28 | 16 | 4 | 0.64 | < LOD | - | - | - | 0.90 | 2.05 |  |
| **CAR** | 159 | 23 | 6.38 | 559 | 44 | 22.44 | 29 | 3 | 0.20 | N | 20.36 | 72.24 |  |
| **MIA** | < LOD | - | - | < LOD | - | - | < LOD | - | - | - | - | - |  |
| **FLU** | 4 | 2 | 0.16 | 204 | 36 | 8.19 | 164 | 27 | 1.15 | N | 0.51 | 29.78 |  |
| **LOR** | < LOD | - | - | < LOD | - | - | < LOD | - | - | - | - | - |  |
| **SAL** | < LOD | - | - | < LOD | - | - | < LOD | - | - | - | - | - |  |
| **WWTP5** | | | | | | | | | | | | | |
| **IBU** | 22 | 6 | 0.88 | < LOD | - | - | < LOD | - | - | 100 | 4.40 | - |  |
| **NAP** | 229 | 9 | 9.16 | < LOD | - | - | < LOD | - | - | 100 | 45.80 | - |  |
| **DIC** | 14 | 4 | 0.56 | 100 | 28 | 4.00 | < LOD | - | - | N | 2.80 | 20.00 |  |
| **KET** | 639 | 42 | 25.56 | < LOD | - | - | < LOD | - | - | 100 | 127.8 | - |  |
| **SUL** | 393 | 19 | 15.72 | 392 | 17 | 15.68 | 3.76 | 0.23 | 0.02 | 0.1 | 78.60 | 78.52 |  |
| **ATE** | < LOD | - | - | < LOD | - | - | < LOD | - | - | - | - | - |  |
| **PRO** | < LOD | - | - | < LOD | - | - | < LOD | - | - | - | - | - |  |
| **MET** | 87 | 3 | 3.48 | 166 | 12 | 6.64 | 8.50 | 0.89 | 0.06 | N | 17.40 | 33.48 |  |
| **FUR** | 76 | 10 | 3.04 | < LOD | - | - | < LOD | - | - | 100 | 15.20 | - |  |
| **CAR** | 215 | 18 | 8.60 | 432 | 23 | 17.28 | < LOD | - | - | N | 43.00 | 86.40 |  |
| **MIA** | < LOD | - | - | < LOD | - | - | < LOD | - | - | - | - | - |  |
| **FLU** | 9 | 5 | 0.36 | 685 | 78 | 27.40 | 392 | 20 | 2.55 | -N | 1.80 | 149.73 |  |
| **LOR** | < LOD | - | - | < LOD | - | - | < LOD | - | - | - | - | - |  |
| **SAL** | < LOD | - | - | < LOD | - | - | < LOD | - | - | - | - | - |  |
| **WWTP6** | | | | | | | | | | | | | |
| **IBU** | 35 | 9 | 5.78 | 289 | 19 | 47.69 | < LOD | - | - | N | 7.40 | 61.13 |  |
| **NAP** | < LOD | - | - | < LOD | - | - | < LOD | - | - | - | - | - |  |
| **DIC** | 3 | 2 | 0.50 | 185 | 29 | 30.53 | < LOD | - | - | N | 0.63 | 39.13 |  |
| **KET** | 496 | 38 | 81.84 | 171 | 17 | 28.22 | < LOD | - | - | 66 | 104.9 | 36.17 |  |
| **SUL** | 117 | 8 | 19.31 | 116 | 15 | 19.14 | < LOD | - | - | 1 | 24.75 | 24.54 |  |
| **ATE** | < LOD | - | - | < LOD | - | - | < LOD | - | - | - | - | - |  |
| **PRO** | < LOD | - | - | < LOD | - | - | < LOD | - | - | - | - | - |  |
| **MET** | 171 | 15 | 28.22 | 204 | 16 | 33.66 | 16 | 1 | 0.67 | N | 36.17 | 44.01 |  |
| **FUR** | 157 | 23 | 25.91 | 495 | 35 | 81.68 | < LOD | - | - | N | 33.21 | 104.7 |  |
| **CAR** | 624 | 76 | 103.0 | 1555 | 146 | 256.6 | 34 | 2 | 1.42 | N | 132.0 | 330.8 |  |
| **MIA** | < LOD | - | - | < LOD | - | - | 161 | 1 | 6.73 | - | - | 8.63 |  |
| **FLU** | 62 | 4 | 10.23 | 508 | 117 | 83.82 | 406 | 20 | 16.98 | N | 13.12 | 129.2 |  |
| **LOR** | < LOD | - | - | < LOD | - | - | < LOD | - | - | - | - | - |  |
| **SAL** | < LOD | - | - | < LOD | - | - | < LOD | - | - | - | - | - |  |

**Table H.** **Environmental risk assessment (RQ) of pharmaceuticals in the effluent of individual WWTPs.**

| **Name of substance** | **Algae** | **Daphnia** | **Fish** | **Algae** | **Daphnia** | **Fish** | **Algae** | **Daphnia** | **Fish** |
| --- | --- | --- | --- | --- | --- | --- | --- | --- | --- |
|  | **WWTP1** | | | **WWTP2** | | | **WWTP3** | | |
| **IBU** | 0.161 | 0.072 | 0.129 | nd | nd | nd | 0.054 | 0.024 | 0.043 |
| **NAP** | nd | nd | nd | nd | nd | nd | nd | nd | nd |
| **DIC** | 0.009 | 0.006 | nd | 0.018 | 0.012 | nd | 0.059 | 0.039 | 0.002 |
| **KET** | 0.583 | 0.003 | nd | 0.458 | 0.002 | nd | 0.075 | nd | nd |
| **SUL** | 0.001 | 0.001 | nd | 0.012 | 0.012 | 0.001 | nd | nd | nd |
| **ATE** | nd | nd | nd | nd | nd | nd | nd | nd | nd |
| **PRO** | nd | nd | nd | nd | nd | nd | nd | nd | nd |
| **MET** | 0.001 | 0.002 | nd | nd | nd | nd | 0.003 | 0.007 | 0.001 |
| **FUR** | nd | 0.001 | nd | nd | 0.001 | nd | nd | nd | nd |
| **CAR** | 0.004 | 0.004 | 0.009 | 0.012 | 0.014 | 0.029 | 0.010 | 0.011 | 0.024 |
| **MIA** | nd | nd | nd | nd | nd | nd | nd | nd | nd |
| **FLU** | 2.833 | 4.443 | 1.333 | 0.814 | 1.276 | 0.383 | 0.508 | 0.796 | 0.239 |
| **LOR** | nd | nd | nd | nd | nd | nd | 4.551 | 1.570 | 10.619 |
| **SAL** | nd | nd | nd | nd | nd | nd | nd | nd | nd |
|  | **WWTP4** | | | **WWTP5** | | | **WWTP6** | | |
| **IBU** | 0.022 | 0.010 | 0.018 | nd | nd | nd | 0.072 | 0.032 | 0.058 |
| **NAP** | nd | nd | nd | nd | nd | nd | nd | nd | nd |
| **DIC** | 0.015 | 0.010 | nd | 0.007 | 0.005 | nd | 0.013 | 0.008 | nd |
| **KET** | 0.192 | 0.001 | nd | nd | nd | nd | 1.425 | 0.007 | nd |
| **SUL** | 0.005 | 0.004 | nd | 0.005 | 0.005 | nd | 0.001 | 0.001 | nd |
| **ATE** | nd | nd | nd | nd | nd | nd | nd | nd | nd |
| **PRO** | nd | nd | nd | nd | nd | nd | nd | nd | nd |
| **MET** | 0.002 | 0.005 | 0.001 | 0.001 | 0.003 | 0.000 | 0.001 | 0.003 | nd |
| **FUR** | nd | nd | nd | nd | nd | nd | 0.003 | 0.008 | 0.001 |
| **CAR** | 0.007 | 0.007 | 0.016 | 0.005 | 0.006 | 0.012 | 0.018 | 0.020 | 0.044 |
| **MIA** | nd | nd | nd | nd | nd | nd | nd | nd | nd |
| **FLU** | 0.255 | 0.400 | 0.120 | 0.856 | 1.343 | 0.403 | 0.635 | 0.996 | 0.299 |
| **LOR** | nd | nd | nd | nd | nd | nd | nd | nd | nd |
| **SAL** | nd | nd | nd | nd | nd | nd | nd | nd | nd |

nd- not determined
